# Gene transfer of wild-type apoA-I and apoA-I Milano reduce atherosclerosis to a similar extent

**DOI:** 10.1186/1475-2840-6-15

**Published:** 2007-05-02

**Authors:** Corinna Lebherz, Julio Sanmiguel, James M Wilson, Daniel J Rader

**Affiliations:** 1 Department of Pathology and Laboratory Medicine, Gene Therapy Program, University of Pennsylvania School of Medicine, Philadelphia, PA, USA; 2 Department of Medicine, Pharmacology, Pathology and Laboratory Medicine, Institute for Translational Medicine and Therapeutics, University of Pennsylvania School of Medicine, Philadelphia, PA, USA; 3 Department of Cardiology, Ludwig Maximilian University, Munich, Germany

## Abstract

**Background:**

The atheroprotective effects of systemic delivery of either apolipoprotein A-I (wtApoA-I) or the naturally occurring mutant ApoA-I Milano (ApoA-I_M_) have been established in animal and human trials, but direct comparison studies evaluating the phenotype of ApoA-I or ApoAI-Milano knock-in mice or bone marrow transplantated animals with selectively ApoA-I or ApoAI-Milano transduced macrophages give conflicting results regarding the superior performance of either one. We therefore sought to compare the two forms of apoA-I using liver-directed somatic gene transfer in hypercholesterinemic mice – a model which is most adequately mimicking the clinical setting.

**Methods and results:**

Vectors based on AAV serotype 8 (AAV2.8) encoding wtApoA-I, ApoA-I_M _or green fluorescent protein (GFP) as control were constructed. LDL receptor deficient mice were fed a Western Diet. After 8 weeks the AAV vectors were injected, and 6 weeks later atherosclerotic lesion size was determined by aortic *en face *analysis. Expression of wtApoA-I reduced progression of atherosclerosis by 32% compared with control (p = 0.02) and of ApoA-I_M _by 24% (p = 0.04). There was no significant difference between the two forms of ApoA-I in inhibiting atherosclerosis progression.

**Conclusion:**

Liver-directed AAV2.8-mediated gene transfer of wtApoA-I and ApoA-I_M _each significantly reduced atherosclerosis progression to a similar extent.

## Background

Apolipoprotein A-I (wtApoA-I) is the primary protein component of high density lipoproteins (HDL) [1] and like HDL cholesterol is inversely associated with atherosclerotic cardiovascular disease. Transgenic overexpression of wtApoA-I in liver substantially reduces progression of atherosclerosis in mice [2,3] and rabbits [4]. Furthermore, somatic gene transfer of wtApoA-I to liver using adenoviral vectors reduces progression [5,6] and induces regression [7] of atherosclerosis.

ApoA-I Milano (ApoA-I_M_) is a rare, naturally-occurring Arg173Cys point mutation in ApoA-I. Heterozygosity for ApoA-I_M _is associated with very low levels of HDL-C but no apparent increased risk of CHD [8]. ApoA-I_M _has been studied extensively with regard to effects on atherosclerosis, including infusion and genetic expression in animals [8,9] and even in a clinical trial of repeated intravenous infusion of an a ApoA-I_M _/phospholipid complexes in patients with CHD [10]. It has been suggested that ApoA-I_M _may be a gain-of-function mutation. Indeed, a recent study perfomed by Shan did show a superior atheroprotective effect of ApoA-I_M _compared to wtApoA-I transduced macrophages after bone marrow transplantation into ApoAI/ApoE double knock-out mice [11]. In contrast to that, female ApoA-I_M _knock-in animals are characterized by higher serum cholesterol levels and the development of larger atherosclerotic lesions compared to ApoA-I knock-in mice [12]. Due to the major differences in outcome of these studies and the lack of resemblance to the clinical setting in which patients with normal ApoA-I levels are treated with a systemically delivered ApoA-I or ApoA-I_M _mimetic compound, we wanted to elucidate the effects of wtApoA-I or ApoA-I_M _overexpression in a mouse model susceptible for atherosclerosis.

Adeno-associated viral (AAV) vectors have been shown to be capable of stable gene transfer and hepatic expression [13,14]. First-generation AAV vectors have been used to express wtApoA-I in mouse liver [15] and ApoA-I_M _in mouse muscle [16], but in both cases the levels of expression and plasma concentrations were extremely low compared with normal ApoA-I levels in mice and humans. Much greater expression can be obtained using vectors based on AAV serotype 8 [17]. A comparative study of AAV serotypes delivered intraportally showed that AAV8-based vectors achieved the highest levels of hepatic transgene expression and ranged from 16 to 110 times greater than that of AAV2; gene transfer from AAV2/7 was intermediate [18].

Therefore, in the current study we used AAV8-based vectors to express wtApoA-I and ApoA-I_M _in LDLreceptor deficient mice to test whether AAV-based overexpression of ApoA-I would reduce progression of atherosclerosis in this model and whether wtApoA-I and ApoA-I_M _differed in their effects on atherosclerosis.

## Methods

### Vectors

Transgenes were human wtApoA-I and human apoA-I-Milano for the treatment groups and green fluorescent protein (eGFP) for the control group. The human ApoA-I-Milano gene was cloned via site directed mutagenesis of the wtApoA-I plasmid by PCR. Correct cloning was confirmed by sequencing and restriction digests. To produce AAV vectors encapsidated in an AAV8 capsid (AAV2.8), a pseudotyping strategy was performed as reported [18,19]. Vectors were purified using a standard cesium sedimentation method and titers were determined via TaqMan analysis using probes and primers targeting the BGH poly(A+) region of the vectors.

### Animal studies

LDL receptor deficient mice on C57BL/6 background (LDLR-/- mice) were purchased from the Jackson Laboratory (Bar Harbor, ME, USA) and maintained as a breeding colony. Mice were given unrestricted access to water. Male mice were put on western diet (0.15% cholesterol, 21% butterfat, DYETS, PA) for 8 weeks. After this time period, one group of animals was killed to determine the amount of atherosclerosis development at baseline. The remaining animals were randomly divided into 3 groups and injected intraportally with AAV2.8-TBG-wtApoA-I, AAV2.8-TBG-ApoA-I_M_, or AAV2.8-TBG-GFP at a dose 1 × 10e12 genome copies/mouse, tail vein injection. Animals remained on a western diet for 6 weeks thereafter and were then sacrificed. From all animals blood was obtained at least two times before and at designated time-points after gene transfer. At time of necropsy livers, hearts and aortas were harvested for further analysis. All studies were approved by the Institutional Animal Care and Use Committee of the Wistar Institute and the University of Pennsylvania.

### Serum analysis

Blood samples were obtained from the retro-orbital plexus. For selected time points serum lipoproteins were separated by Fast Performance Liquid Chromatography (FPLC). The amount of plasma lipoproteins in the serum or the FPLC fractions was detected using an automated clinical chemistry analyzer (Alpha Wassermann, USA) or a manual assay according to the manufacturers instruction (Wako, USA). FPLC fractions #3;-#10 are corresponding to VLDL, #11;-#24 to IDL/LDL, and #25;-#40 to HDL.

### Western Blot

Serum (0.25 μl/well) or FPLC fractions (5 μl/well) were resolved on a 10% BisTris gel and transferred to PVDF membrane (Invitrogen, Carlsbad, California, USA). A polyclonal goat anti-human ApoA-I antibody (K45252G, Biodesign, Maine, USA) was used as primary antibody. Blots were developed using chemiluminescence.

### RealTime PCR

Gene expression was analyzed in liver samples using the "Assay on demand" System for murine and human apolipoprotein A-I from Applied Biosystems (Foster City, CA, USA) according to the manufacturer's instructions.

### Histological analysis

For the quantification of the aortic plaques, the mouse aorta was carefully harvested and stained with Sudan IV (Sigma, Germany). Plaque size was determined using Phase 3 Imaging System (Media Cybernetics).

### Statistical analysis

For the statistical analysis a T-Test was performed. Data are described as Mean ± standard error of the mean (SEM). Differences with a p-value less than 0.5 were considered significant.

## Results

### ApoA-I expression and plasma lipids

**Figure 1:**
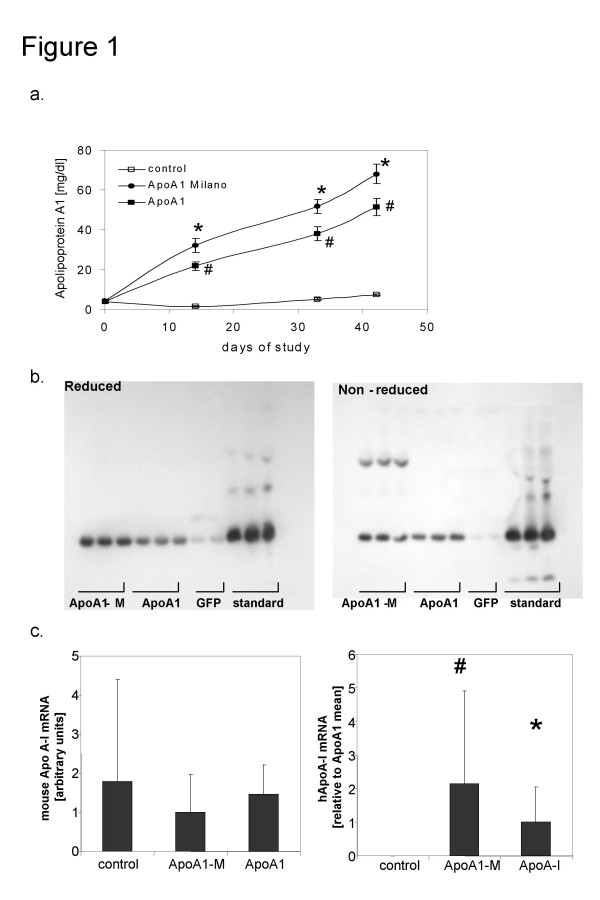
a) Serum levels of human apoA-I as evaluated at designated time points over the course of the study. There was no significant cross-reaction between mouse and the human apoA-I. Human apoA-I levels were increased in the groups treated with the human wtApoA-I (* p < 0.01) and ApoA-IM (# p < 0.01) compared to control b) Western Blots of mouse serum obtained 14 days after gene transfer under reduced and non-reduced conditions. In contrast to animals treated with the wtApoA-I or GFP-control vector there was under non-reduced conditions an additional band detected in the samples of the ApoA-IM animals corresponding with an apoA-I dimer. c) RealTime PCR from liver RNA for murine and human ApoA-I mRNA levels. There was no significant difference in expression levels of murine ApoA-I. Human ApoA-I was only detected in treated animals without a difference in mRNA levels between animals receiving the wild type or the mutant ApoA-I.

Two weeks after injection, plasma levels of human ApoA-I were about 20 mg/dL in mice expressing wild-type wtApoA-I and about 30 mg/dl in mice expressing ApoA-I_M_, and they increased during the 6 weeks (Fig 1a and Table 1). Non-reduced SDS-PAGE of plasma revealed the presence of ApoA-I dimers in the ApoA-I_M _but not in the wtApoA-I treated or control groups (Fig 1b). At six weeks, upon necropsy, the hepatic mRNA abundance in the wtApoA-I and ApoA-I_M _groups were not significantly different (Fig 1c).

**Table 1:** Lipid profiles 6 weeks after gene transfer (14 weeks on Western Diet).

	control	ApoA-I Milano	wtApoA-I
Cholesterol [mg/dl]	1843 ± 121	1844 ± 107	1873 ± 143
Triglycerides [mg/dl]	480 ± 80	617 ± 63	531 ± 88
HDL [mg/dl]	319 ± 15	248 ± 19 **	272 ± 17 *
Phospholipids [mg/dl]	1035 ± 64	1002 ± 63	992 ± 66
Human ApoA-I [mg/dl]	7.5 ± 0.4	68 ± 4.8 **,#	51 ± 4,3 **

** p < 0.01 vs. control, * p < 0.05 vs. control, # p < 0.05 vs. ApoA-I

**Figure 2:**
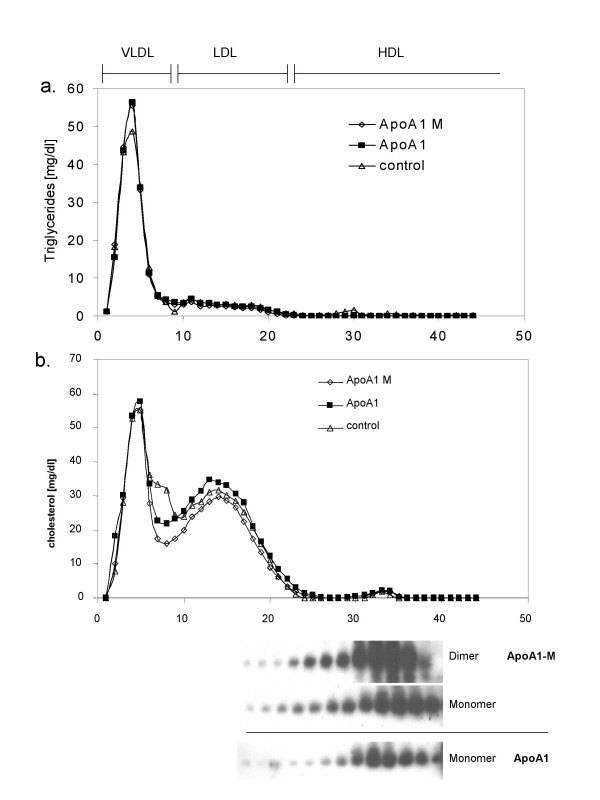
Cholesterol and triglyceride analysis of FPLC fractions of serum samples 14 days after viral gene transfer. Western blot analysis for human apoA-I in the HDL fractions is shown below the cholesterol tracing.

Expression of wild-type wtApoA-I or ApoA-I_M _had no significant effect on plasma total cholesterol, triglycerides, or phospholipids, but did reduce HDL-C levels (Table 1). Lipoprotein fractionation revealed no significant differences in the distribution of cholesterol among the three groups (Fig 2a). Western Blot analysis of the HDL fractions revealed both monomer and dimer of ApoA-I_M _and only monomer of wtApoA-I, as expected and showed no significantly different distribution pattern of the ApoA-I protein between the ApoA-I_M _and the wtApoA-I treated animals (Fig 2b).

### Atherosclerosis progression

**Figure 3:**
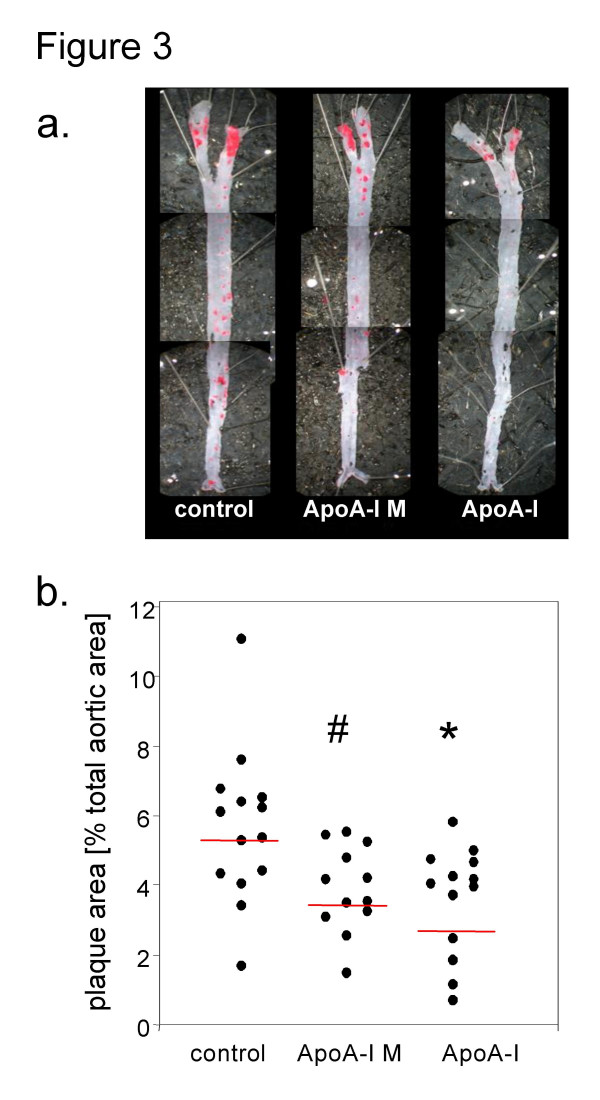
a) Representative aortas stained with Sudan IV. b) Determination of total aortic plaque area as evaluated with *en face *analysis. Compared with the control group, there was significantly less atherosclerosis animals treated with wtApoA-I (*p < 0.02) and ApoA-IM (# p < 0.04) but no significant difference between the wtApoA-I and ApoA-IM groups.

All three groups of mice injected with vector and followed for an additional 6 weeks had more atherosclerosis than mice analyzed at baseline (Fig. 3b). Animals injected with AAV8-wtApoA-I had 32% less aortic atherosclerosis than control animals (p = 0.02), whereas animals injected with AAV8-ApoA-I_M _had 24% less atherosclerosis than controls (p = 0.04) (Figs 3a and 3b). There was no statistical difference between the wild-type and ApoA-I_M _groups.

## Discussion

In this manuscript, we report two new findings. First, we demonstrate using an AAV8-based vector to express wild-type human ApoA-I in the livers of atherosclerosis-prone mice that expression of ApoA-I is of sufficient extent and duration to significantly slow the progression of atherosclerosis. Second, in a direct comparison, we demonstrate that while expression of ApoA-I_M _also significantly slowed progression of atherosclerosis compared with controls, it did not differ significantly from wtApoA-I with regard to effects on atherosclerosis.

While somatic gene transfer of wtApoA-I to liver using adenoviral vectors has been shown to reduce progression [5,6] and even induce regression [7] of atherosclerosis in mice, expression is transient and associated with substantial inflammation. In contrast, expression of wtApoA-I using these AAV8-based vectors was stable for 6 weeks and was associated with no increases in liver transaminases (data not shown). Importantly, expression and plasma levels of wtApoA-I were substantially higher than were seen using first generation AAV vectors [15]. This allowed us to reliably compare two different forms of ApoA-I, wild-type and Milano, with regard to their effects on atherosclerosis.

Previous studies have already evaluated the effects of bone marrow transplantations with retrovirally ApoA-I or ApoA-I_M _transduced macrophages into hyperlipidemic ApoA-I/ApoE double knock out mice. In this experimental setting selective overexpression of wtApoA-I or ApoA-I_M _in macrophages significantly decreased aortic atherosclerosis as well as atherosclerotic plaque macrophage immunoreactivity, effects which were more pronounced with ApoA-I_M _compared to wtApoA-I [11]. In a second study the phenotype of ApoA-I or ApoA-I_M _knock-in mice on an atherosclerosis prone ApoB/ApoAII background was analyzed [12] with the finding of a less favorable lipid profile and a more pronounced atherosclerosis development in female ApoA-I_M _knock-in mice. These results provided by Shah and Rubin's groups provide cardinal differences in the comparison of wtApoA-I or ApoA-I_M_, which might be due to the selection of animal models.

Nevertheless, both studies lack resemblance to the clinical setting in which patients with normal ApoA-I levels are treated with a systemically delivered ApoA-I or ApoA-I_M _mimetic compound. In our current report, we demonstrate that AAV8-mediated gene transfer with hepatic expression of wtApoA-I or ApoA-I_M _results in plasma levels of ApoA-I that are sufficient to significantly slow the development of atherosclerosis in mice. Interestingly we find that both forms, wtApoA-I and ApoA-I_M_, to be similar effective in their ability to retard atherosclerosis development in LDLr knockout mice.

Atheroprotection did occur despite a reduction in serum HDL levels in both treatment groups. This mimics the human situation, in which ApoA-I Milano carriers have reduced levels of HDL cholesterol, which paradoxically is associated with atheroprotection.

The exact mechanism for the ApoA-I Milano induced decrease in HDL levels is not completely elucidated yet and the fact, that so far only heterozygotes for the mutation have been identified, who are producing wild-type and mutated ApoA-I, is making the answer more difficult. One of the hypotheses is that HDL-bound ApoA-I Milano leads to a faster catabolism of the HDL particles. This increase in cholesterol efflux from HDL-ApoA-I Milano particles was proven in ApoA-I Milano transgenic mice, which also display a decrease in HDL levels [20,21].

The lipid data obtained in our animal model are therefore highly consistent with the human phenotype and the results from the transgenic animals. In our model HDL levels did also slightly decrease in those animals treated with wtApoA-I, even though to a lesser extent than in those treated with ApoA-I Milano. Hepatic mRNA levels of murine ApoA-I did not differ between control and wtApoA-I or ApoA-I Milano treated animals in our study, therefore a down-regulation of the endogenous gene due to the AAV gene transfer is unlikely.

It is clear that the plasma HDL concentration itself is neither a surrogate for its functionality nor the kinetics of reverse cholesterol transport [22]. Since the main determinant of HDL levels is its clearance and the metabolism of the mature HDL particle is highly dependent on its composition, it is imaginable that also the wtApoA-I gene transfer did led to the production of HDL particles with a more favorable metabolism.

Another assumption is, that essentially replacing murine apoA-I with human apoA-I in these studies resulted in a more functional HDL that inhibited atherosclerosis, and that wild-type and Milano did not differ in this effect. Indeed, there is substantial interest in the structural differences between human and murine apoA-I and it is plausible that they would differ in their anti-atherogenic effects. Further experiments have to be performed to further elucidate this phenomenon.

In conclusion, these results support the concept of using second generation AAV8-based vectors to achieve hepatic gene transfer and expression of transgenes that encode abundant plasma proteins (such as ApoA-I) for studies in animals. They also suggest that systemic wtApoA-I overexpression is as effective as ApoA-I_M _overexpression in reducing atherosclerosis progression in susceptible mice. Finally, they suggest that AAV8-mediated gene transfer of either wtApoA-I or ApoA-I_M _could be considered as an experimental clinical approach for the treatment of atherosclerosis.

## Competing interests

This study was supported by P01 HL59407 and P01 HL22633 from the National Heart Lung and Blood Institute.

## Authors' contributions

JS analyzed the tissues via RealTime PCR for gene expression, JMW and DJR participated in the study design and coordination and helped to critically review and draft the manuscript. All authors read and approved the final manuscript.
